# Perceived challenges and opportunities arising from integration of mental health into primary care: a cross-sectional survey of primary health care workers in south-west Ethiopia

**DOI:** 10.1186/1472-6963-14-113

**Published:** 2014-03-06

**Authors:** Mubarek Abera, Markos Tesfaye, Tefera Belachew, Charlotte Hanlon

**Affiliations:** 1 Department of Psychiatry, College of Public Health and Medical Sciences, Jimma University, PO Box 378, Jimma, Ethiopia; 2 Population and Family Health Department, College of Public Health and Medical Sciences, Jimma University, PO Box 1104 Jimma, Ethiopia; 3 Department of Psychiatry, School of Medicine, College of Health Sciences, Addis Ababa University, PO Box 9086, Addis Ababa, Ethiopia; 4 King’s College London, Institute of Psychiatry, Health Services and Population Research Department, London, UK

**Keywords:** Mental health, Mental disorders, Mental health care, Primary health care, Task shifting, Scale-up, Sub-Saharan Africa, Low-income country

## Abstract

**Background:**

The WHO’s mental health Gap Action Programme seeks to narrow the treatment gap for mental disorders by advocating integration of mental health into primary health care (PHC). This study aimed to assess the challenges and opportunities of this approach from the perspective of PHC workers in a sub-Saharan African country.

**Methods:**

A facility-based cross-sectional survey of 151 PHC workers was conducted from 1^st^ to 30^th^ November 2011 in Jimma zone, south-west Ethiopia. A structured questionnaire was used to ask about past training and mental health experience, knowledge and attitudes towards mental disorders and provision of mental health care in PHC. Semi-structured interviews were carried out with 12 heads of health facilities for more in-depth understanding.

**Results:**

Almost all PHC workers (96.0%) reported that mental health care was important in Ethiopia and the majority (66.9%) expressed interest in actually delivering mental health care. Higher levels of general health training (degree vs. diploma) and pre-service clinical exposure to mental health care were associated with more favourable attitudes. Knowledge about mental disorder diagnoses, symptoms and treatments was low. Almost half (45.0%) of PHC workers reported that supernatural factors were important causes of mental disorders. Health system and structural issues, such as poor medication supply, lack of rooms, time constraints, absence of specialist supervision and lack of treatment guidelines, were identified as challenges. Almost all PHC workers (96.7%) reported a need for more training, including a clinical attachment, in order to be able to deliver mental health care competently.

**Conclusions:**

Despite acceptability to PHC workers, the feasibility of integrating mental health into PHC in this sub-Saharan African setting is limited by important gaps in PHC worker knowledge and expectations regarding mental health care, coupled with health system constraints. In addition to clinically-based refresher mental health training, expansion of the specialist mental health workforce may be needed to support integration in practice.

## Background

Mental, neurological and substance use (MNS) disorders are prevalent in all regions of the world and are major contributors to morbidity and premature mortality [1]. MNS disorders alone contribute 10.4% of the total global burden of disease, mostly accounted for by depression and other common mental disorders, alcohol and substance-use disorders, psychoses and epilepsy [2]. Mental disorders are at least as disabling as cancer or heart disease in terms of lost productivity and premature death [3]. In Ethiopia, schizophrenia and depression are ranked in the top ten contributors to disease burden [4]. Despite this, the amount of money spent on mental health care in low- and middle-income countries (LMICs) is disproportionately low when compared to physical health problems [5]. Evidence-based treatments for MNS disorders are available and have been shown to be appropriate for LMIC settings [6]. However, the availability of effective mental health care does not match the burden of MNS disorders, leading to a large treatment gap: in LMICs up to 90% of persons with severe mental disorders go untreated [7], compared to less than 50% in high-income countries [8]. The consequences of untreated mental disorders in LMICs are substantial, associated with personal suffering, premature mortality [9], poorer physical health and health care [3], poverty [10], caregiver burden [11], stigma, discrimination and abuse [12].

Specialist mental health workers are scarce in LMIC settings and cannot, in isolation, provide the solution to the MNS disorder treatment gap [13]. In Ethiopia, there is about one psychiatrist per two million people [14] and only an estimated 0.68 mental health professionals per 100,000 people [15], largely concentrated in urban areas and hospital settings which are not accessible to the majority of the population. Integration of mental health care into primary health care (PHC), through a model of task sharing with mental health specialists, is therefore advocated as the best approach to narrow the treatment gap for MNS disorders [1]. Providing mental health care in the PHC setting has other potential benefits, including more holistic care and better physical health outcomes, reduced stigma and more locally available care with a resultant decrease in the economic burden of care and a greater chance of maintaining contact with community and family support systems [16]. Furthermore, co-morbidity between physical and mental health conditions is high [3] and the expression of mental health problems as physical symptoms (somatisation) is widespread (and often undetected) in general health care settings [17]. However, despite the desirability of task sharing from a public mental health and health service planning perspective, little is known about the perspectives of frontline PHC workers on taking on these new mental health care roles. A recent systematic review of the acceptability and feasibility of task sharing mental health care with PHC workers in LMICs found that studies were scarce and were often concerned with clearly circumscribed interventions for specific disorders rather than providing a general mental health service [18].

This paper contributes to our understanding of the perspectives of PHC workers towards mental health care in a sub-Saharan African setting characterized by a high burden of infectious diseases and under-nutrition and over-stretched PHC services. Such information is essential in order to meet the training and support needs of PHC workers and contribute to the successful scale up of mental health care. The aim of the study was, therefore, to assess the perceived challenges and opportunities for integrating mental health care into a primary health care from the perspective of PHC workers in a regional zone in Ethiopia.

## Methods

### Setting

The study was conducted in Jimma zone of the Oromiya region, located in south west Ethiopia. Based on the 2007 census, this zone has a total population of 2,486,155 people [19]. The zone is predominantly rural, with most inhabitants living off the land. Jimma zone is where the largest coffee plantations in Ethiopia are located. Other crops include cereals, pulses, root vegetables and fruit, with maize and barley grown in the highlands and swamp areas [19].

### Health system context

In Jimma zone, and across Ethiopia, the PHC unit comprises a health centre, usually staffed by health officers, midwives and clinical nurses (degree and diploma level), satellite health posts and community-based health extension workers (HEWs). In Jimma zone there are 56 health centres and 472 health posts. The potential health service coverage by health post and health centre is estimated to be 92% and 43% of the population, respectively [20]. The main focus of PHC services is preventive care, maternal and child health, and treatment of infectious diseases. Most health centres offer mainly out-patient services, with some in-patient provision for emergency care and deliveries. The duration of training for PHC staff is as follows: health officers (three to four years, depending on year of graduation), degree-level clinical nurses (three to four years, depending on year of graduation), diploma level clinical nurses (three years), diploma level midwives (three years), HEWs (one year). Health officers and nurses are responsible for providing clinical care in the health centre setting, including prescribing medications where needed, as there are very few medical doctors practicing in PHC.

In terms of mental health care, there is one psychiatric unit in Jimma zone, staffed by two psychiatrists and ten non-psychiatrist mental health professionals. This unit provides both in-patient and out-patient services. Outside of this specialist unit there is no provision of mental health care within the zone.

### Study design

A quantitative cross-sectional survey augmented by semi-structured interviews.

### Sample

Out of the 56 health centres in Jimma zone, 16 health centres were selected using simple random sampling (lottery method). Each health centre had an equal chance of being selected. All health officers, clinical nurses and midwives working in these selected health centres were approached to participate in the study. Only those PHC workers who were working on the day of the survey were eligible for the study.

### Measures

Data were collected using a self-completed questionnaire that contained three sections:

(1) Experience and training

Self-report of years of clinical experience and whether or not the respondent had received pre-service or in-service training in mental health care, the duration of training and whether or not the training included a clinical attachment.

(2) Knowledge and Attitudes Questionnaire

A structured questionnaire to investigate knowledge and attitudes of PHC workers towards mental illness and health care was developed for the purpose of the project. The questionnaire drew on previous research in this area from LMIC settings [21-23] as well as taking into account the mental health teaching curriculum for general health workers in Ethiopia. The knowledge section used open-ended structured questions to ask respondents to list mental illnesses and psychotropic medication. The question on possible causes of mental illnesses allowed respondents to endorse more than one cause.

(3) Health facility factors

Questions were asked about the availability of psychotropic medications, rooms for consultation, guidelines for treatment of mental illnesses, consultation and supervision opportunities for mental health care, as well as time to deliver mental health care in the health facility in which the respondent was working.

The initial English version of the questionnaire was translated to Amharic and then back-translated into English independently to check for consistency and semantic validity.

### Data collection

Data collection was carried out from 1^st^ to 30^th^ November 2011 by two diploma level professionals who were fluent in Amharic and English. Two trained health officers were assigned as supervisors throughout the study period. The supervisors checked the questionnaires for completeness and were available to solve any problems that arose during data collection.

To illuminate understanding of the quantitative study findings, twelve in-depth interviews were carried out with the heads and technical heads of participating health centres using a semi-structured interview guide. The topics covered by the guide included the respondent’s views about the importance and relevance of integrating mental health care into primary care, the existing situation and some of the logistical challenges that would be faced in integrating mental health care. The interviews were carried out by two Bachelor degree level professionals with exposure to qualitative methodology but limited experience in qualitative data collection. Notes were taken during the discussions, in addition to audio-taping, in order to minimise loss of information. The audiotape was transcribed into a text document which was translated into English.

### Data quality assurance

The structured questionnaire was pre-tested to ensure acceptability and comprehensibility. The data collectors were trained for one and a half days, focusing on the materials, methods and administration of the instruments. Data was checked at the field level by the investigator. Epi-data version 3.1 was used for double-data entry to minimize error during entry.

### Data analysis

The data were cleaned before final analyses by looking at the distribution of the data, identifying outliers and checking back against the original data. Data analysis was carried out using SPSS version 16 for Windows. Most of the responses to the structured questionnaire were analysed descriptively, as percentages or summary measures of central tendency. Univariate analyses of the associations between selected responses and respondents’ socio-demographic features (e.g. qualification, gender and sex) were investigated using a *χ*^2^ test.

The qualitative data were coded and categorized by the first author using a thematic analysis approach. For the purposes of this paper, verbatim quotes from the semi-structured interviews were used to illustrate the quantitative findings. Quotes were selected to be representative of the main themes and checked back against the data.

### Ethical clearance

Ethical clearance was obtained from the research ethics committee of Jimma University Faculty of Public Health and Medical Science. Informed, written consent was obtained from each study participant. The right to withdraw from the research process at any point in time was respected. Privacy and strict confidentiality was maintained during the interview process.

## Results

### Socio-demographic characteristics

Out of 158 PHC workers eligible for inclusion in the study, 151 actually participated, giving a response rate of 95.6%. The seven non-participating PHC workers were on annual leave or attending in-service training at the time of the study. The respondents comprised 102 (67.5%) clinical nurses with diploma level training, 29 (19.2%) health officers, 14 (9.3%) nurses with degree level training and six (4.0%) diploma level trained midwives. In the total sample, 55.6% (n = 84) of respondents were female and 57.6% (n = 87) were single. In terms of religious affiliation, 48.3% (n = 73) of respondents were Orthodox Christians and 38.4% (n = 58) were Muslim. The mean age (standard deviation; SD) of the respondents was 28.6 (SD 6.17) years. See Table 1.

**Table 1:** Characteristics of respondents (n = 151)

**Characteristics**		**N (%)**
Sex	Female	84 (55.6)
Age	(Years)	Mean (SD) 27 (6.18)
Religious affiliation	Orthodox Christian	73 (48.3)
	Muslim	58 (38.4)
	Protestant	15 (9.9)
	Catholic	4 (2.6)
	Others	1 (0.7)
Marital status	Single	87 (57.6)
	Married	55 (36.4)
	Divorced or widowed	8 (5.3%)
Educational level	Diploma	108 (71.5)
	Degree	43 (28.5)
Field of study	Diploma clinical nurse	102 (67.5)
	Health officer	29 (19.2)
	Degree clinical nurse	14 (9.3)
	Midwife	6 (4.0)
Year of work experience	1 – 2	43 (28.5)
	3 – 5	31 (20.5)
	> 5	77 (51.0)
Pre-service training in mental health	Yes	137 (90.7)

### Mental health training

Most of the respondents (90.7%; n = 137) reported that they had received pre-service training in mental health care. However, of these, only 31.4% (n = 43) reported having a clinical attachment in mental health care during their training; 72.1% (n = 31) of degree nurses and 12.8% (n = 12) of diploma nurses. None of the respondents had participated in any in-service training in mental health care since graduating.

### Attitudes towards mental health care and its integration in PHC

Almost all respondents (98.0%; n = 148) expressed a positive attitude towards mental health and the idea of integration of mental health care into PHC services. Most respondents (92.1%; n = 139) reported that mental illness is a problem for Ethiopia and 96.0% (n = 145) expressed the opinion that mental health care is important for the country. Similarly, 91.4% (n = 138) of respondents supported the idea of providing mental health care within their health centre, although a lower percentage (66.9%; n = 101) expressed interest in actually delivering mental health care themselves. Expressed willingness to deliver mental health care was related to level of training: 83.7% (n = 36) of degree-level clinical nurses compared to 60.2% (n = 65) of diploma-level nurses (χ^2^ = 7.96, degrees of freedom (df) = 1, p = 0.005). One in 20 PHC workers (4.6%; n = 7) responded that traditional healers were more effective in treating mental illness than modern medicine, and 2.6% (n = 4) responded that mentally ill persons should not receive mental health care in the health centre setting. Just over one quarter (25.2%) of respondents reported that treating persons with MNS disorders in the health centre would put other patients at risk. See Table 2.

**Table 2:** Primary health care worker attitudes towards mental illness and mental health care (n = 151)

	**Attitude**		
	**Agree N (%)**	**Neutral N (%)**	**Disagree N (%)**
Mental illness is a problem for Ethiopia	139 (92.1)	3 (2.0)	9 (6.0)
Mental health care is important	145 (96.0)	4 (2.6)	2 (1.3)
Some health workers believe that health centres can’t play any role in intervening for mental illness? What about you?	7 (4.6)	11 (7.3)	133 (88.1)
Are you interested to have mental health services integrated into your health centre?	138 (91.4)	8 (5.3)	5 (3.3)
Are you personally interested in actually delivering mental health care in your own health centre?	101 (66.9)	21 (13.9)	29 (19.2)
Some health workers believe that delivering mental health services in the health centres will put other patient in danger? What about you?	38 (25.2)	17 (11.3)	96 (63.6)
Some health workers believe that mentally ill patient should not be treated in the same health centre with the general patient. What about you?	4 (2.6)	9 (6.0)	138 (91.4)
Some health workers believe that traditional healers are better in effectiveness than our medical care? What about you?	7 (4.6)	11 (7.3)	133 (88.1)
As mental illness is not curable, delivering mental health care is wasting resources.	6 (4.0)	5 (3.3)	140 (92.7)

The contrasting views regarding the importance of providing mental health care in PHC are illustrated below in the quotes from two health centre heads:

“Mental illness is a problem for the country as the illness is common in productive age groups as well as substance users; which in turn is becoming a common problem for the youth and adolescents; which leads to loss of productive manpower resulting in adverse economic growth for the patient, his family and the country as a whole”

“It is not as such a big problem for the country as I’m not seeing many patients in my locality”

Pre-service training in mental health care was associated with a more favourable attitude towards the integration of mental health care into PHC services; 92.7% (n = 127) of trained PHC workers expressed a positive attitude compared to 78.6% (n = 11) of those without pre-service training (*χ*^2^ = 8.57, df = 1, p = 0.003). Similarly, those who had experienced clinical attachments during pre-service training had more positive attitudes towards delivering mental health care: 93.0% (n = 40) of those with clinical attachment expressing a positive attitude compared to 56.4% (n = 53) of those without clinical exposure (*χ*^2^ = 14.97, df =1, p < 0.001). There was no association between age, sex, marital status or the religious affiliation of respondents and attitude towards delivering mental health care in PHC.

PHC worker perspectives on the best place to provide mental health care was as follows: health centres (63.6%; n = 96), specialized hospitals (29.8%; n = 45) and general hospitals (6.6%; n = 10). Again there was a difference depending on the level of training, with 76.7% (n = 33) of degree nurses favouring the health centre for delivery of mental health care compared to only 58.3% (n = 63) of diploma nurses (*χ*^2^ = 6.49, df = 2, p = 0.039). In those respondents favouring delivery of mental health care in health centres, the main justifications were accessibility (59.6%; n = 90), affordability (6.0%; n = 9) and acceptability (1.3%; n = 2) for care-seekers. In those respondents favouring hospital-based mental health care, the presence of well trained health workers, including psychiatrists and other specialist mental health professionals (20.5%; n = 31), and better quality of care (10.6%; n = 16) were the reasons given.

When asked about of the scope of mental health care that should be delivered in PHC, the largest percentage of respondents (48.3%; n = 73) identified prevention of mental illness as a potential role for their PHC institution, followed by direct clinical care of patients (34.4%; n = 52), referral of persons with mental illness to higher levels within the health system (29.8%; n = 45) and provision of ongoing follow-up for persons with established mental illness (8.6%; n = 13). Only a small proportion of respondents (4.0%; n = 6) reported that their health institution would have no role in mental health care.

### Knowledge about mental illness and treatment

When asked to list all the mental illnesses that they knew of, 18.5% (n = 28) of respondents did not list any mental illness. The most frequently identified mental illness was depression (60.0%; n = 92) followed by anxiety (41.1%; n = 62), schizophrenia (40.4%; n = 61) and mania or bipolar disorder (27.8%; n = 42).

When asked to list mental disorders and their symptoms, 76.8% (n = 116) did not list any symptoms, indicating a low level of awareness. Of those who listed symptoms of depression, the most frequently identified symptoms were lack of happiness (n = 12), low self-esteem (n = 11), loss of interest (n = 6) and loss of appetite (n = 6). In those who listed symptoms of schizophrenia, 12 respondents identified delusions and eight identified hallucinations as being characteristic symptoms and signs.

Regarding the causes and risk factors for mental illness, 45.0% (n = 68) of the PHC workers considered supernatural or spiritual factors to be important (see Table 3). However, almost all respondents thought that biological (98.0%; n = 148) and psychosocial (92.0%; n = 139) factors played a role. Diploma level PHC workers were significantly more likely to endorse supernatural causes and risk factors for mental illness when compared to degree level workers: (51.9%; n = 56 vs. 27.9%; n = 12); Pearson *χ*^2^ (df = 1) 7.12, p = 0.008.

**Table 3:** Primary health care (PHC) worker knowledge about the causes of, and risk factors for, mental illness (n = 151)

***Reported risk factors***	**n (%)**
***Supernatural***	
Evil eye	34 (22.5)
Evil spirit	20 (13.2)
Attack from the devil	26 (17.2)
Magic	44 (29.1)
Due to sins committed	18 (11.9)
Will of God	20 (13.0)
Curse	31 (20.5)
***Biological***	
Genetic exposure	87 (57.6)
Use of psychoactive substances	144 (95.4)
Neurochemical imbalance	123 (81.5)
***Psychosocial***	
Financial constraint	87 (57.6)
Loss of loved one	86 (57.0)
Academic failure	86 (57.0)
Conflict in marriage	104 (68.9)
Unemployment	86 (57.0)
Divorce	99 (65.6)
Work overload	69 (45.7)
Physical or sexual abuse	120 (79.5)

Most of the respondents (96.7%; n = 146) reported knowing about the existence of effective medication to treat mental illness. However, when asked to identify medications used in mental health care, the majority could not identify either an antipsychotic or antidepressant medication: diazepam was identified by 70.2% (n = 106), chlorpromazine by 33.1% (n = 50) and amitriptyline by 32.5% (n = 49).

PHC workers reported their sources of information about the causes of mental illness to be college or university study (n = 50; 33.1%), public media (n = 38; 25.2%), professional work colleagues (n = 14; 9.3%) and others, e.g. friends.

More than half of respondents (60.3%; n = 89) reported that they were not satisfied with their current level of mental health knowledge, and almost all (96.7%; n = 146) reported that in-service training would be needed for health professionals to improve their knowledge sufficiently to be able to deliver mental health care in the PHC setting. This concern about competence is illustrated in the quote from the health centre head given below:

*“Think, I’m a BSc nurse staff but I can’t assess and examine a person for mental illness as my undergraduate study was not really practical skills teaching; I have only seen patients by my eye but never spoken to them, while the patient was assessed by the teacher. Here, most are diploma level professionals, who never seen a patient during their training, so how can we expect them to diagnose and treat person with mental illness? Therefore, before integration proceeds, skilful in-service training is mandatory for the health workers so as to enable proper assessment and treatment of a person with mental illness”.* Institutional and health system factors

Only 31.8% (n = 48) of respondents reported availability of medications to treat mental illness in their health institution, and where available, this was mostly diazepam (n = 39; 25.8%). Chlorpromazine was only reported to be available by 6.0% (n = 9) of respondents and amitriptyline by 3.3% (n = 5) of respondents. Around half of the respondents (49.7%; n = 75) reported that they would face a shortage of examination rooms if their PHC facility started to deliver mental health care. A similar percentage (45.0%; n = 68) reported that they would not have enough time to deliver mental health care. Nearly all of the respondents reported that they had no formal discussions about mental disorders during teaching sessions with higher level supervisors (99.3%; n = 150) or with other health worker colleagues (96.7%; n = 146). There were no mental health-related guidelines within any of the health centres.

## Discussion

In this cross-sectional survey of frontline PHC workers from a low-income sub-Saharan African country setting, comprehensive information was obtained about knowledge gaps and the acceptability and feasibility of task sharing mental health care with PHC workers.

Whereas almost all of the PHC workers considered MNS disorders to be a problem in Ethiopia, accepted the need for mental health care and supported the integration of mental health care into the health centres where they worked, a somewhat lower percentage (66.9%) expressed interest in actually being involved in the delivery of mental health care themselves. Furthermore, it is noteworthy that the envisaged role of PHC was largely in mental illness prevention, referral to specialists and follow-up of patients seen by specialist mental health workers: the acceptability of a more active role in delivering mental health treatment, for example, as outlined in the WHO’s mhGAP Implementation Guide [24], was less clear. This disconnect between recognising the need to deliver mental health care in PHC and willingness to be personally involved in the delivery of mental health care has been seen in several previous studies [23,25-27]. In a qualitative study in South Africa, primary care-based HIV workers recognised the need for mental health care in the PHC setting but reported that, after screening and referral, this care should be delivered by another professional, preferably a mental health worker [25]. In the seminal WHO studies carried out over 30 years ago to evaluate integration of mental health into PHC in seven LMICs, the findings were much the same: although not overtly opposed to the idea of expanding PHC to include mental health care, the PHC workers expected this to be carried out by mental health workers rather than becoming part of their work [22].

Several factors could be influencing PHC worker willingness to provide mental health care in our study, including their perceived competence, the perceived effectiveness of modern medicine and stigmatizing beliefs about MNS disorders. It is important to note that over a quarter of respondents thought that persons with MNS disorders would pose a risk to other patients. Similar concerns were expressed in a study of PHC workers in Zambia, with the majority of respondents recommending that services for persons with mental illness should be delivered in a separate facility and expressing discomfort about the idea of direct involvement in delivering care to people with mental illness [26]. Concerns about the dangerousness of persons with mental illness have also been found in studies of PHC workers in Nigeria [28]. This indicates that many PHC workers equate MNS disorder with the overt manifestations of mental illness seen in acute psychosis or mania, similar to the findings from a community study in Ethiopia [29]. Training of PHC workers needs to target this misperception, as well as properly addressing the concerns of PHC workers with regard to safe management of the small proportion of persons with MNS disorders who do pose a potential risk to others.

More highly trained health workers were more favourably disposed towards the idea of delivering mental health care and less likely to consider supernatural factors to be important in aetiology of mental disorders, in keeping with findings from a study of PHC workers in Nigeria [27]. PHC workers with pre-service clinical attachments in mental health care were much more likely to express a favourable attitude towards the idea of delivering mental health care. Practical exposure to mental health care has the potential to be more effective in overcoming reluctance to provide care. First-hand contact with persons with mental illness has been shown to be a potent factor in reducing stigmatising attitudes [30]. At present only a small proportion of PHC workers (31.4%) reported that they had been exposed to practical attachments during their training and none had received any in-service or on-the-job training in mental health care. The current lack of mental health specialists in Ethiopia means that there are a limited number of facilities available for training PHC workers. These training facilities are often overloaded with students from a range of disciplines (medical students, health officers, medical interns, psychologists, psychiatric nurse trainees and psychiatric residents) which may limit opportunities for hands-on clinical training. Furthermore, most mental health facilities are hospital-based and concerned with the care of persons with severe mental disorders, usually psychosis, whereas PHC workers also need to develop skills in detecting and managing the common mental disorders (for example, depression, anxiety and alcohol use disorders). This serves to underline the importance of expanding the specialist mental health workforce and developing their role as trainers in order to be able to support scale up of mental health care integrated into PHC settings [1,31].

The general level of knowledge about MNS disorders and pharmacological treatments was low in this sample of PHC workers. Depression and anxiety were the most frequently reported MNS disorders (reported by 60.0% and 41.1%, respectively). However, when asked to list symptoms that are characteristic of the disorders, no PHC workers identified the common presenting symptoms of depression in the Ethiopian PHC setting, i.e. headache, non-specific aches and pains and fatigue [32,33]. This may indicate a more theoretical rather than practical knowledge of depression and anxiety arising from a lack of clinical exposure during training. The context of PHC in Ethiopia also needs to be considered in this regard. The number of patients seen by PHC workers per day is very high which might mitigate against acquisition of detailed knowledge about symptoms of depression and anxiety, akin to findings from training of general doctors in India [34]. Training will need to emphasise more common and easily identifiable presentations of depression and anxiety for the setting. When asked about the possible causes of MNS disorders, more than half of the PHC workers reported genetic predisposition and neurochemical imbalance as risk factors for MNS disorders, which is similar to a study of PHC workers in Nepal [23]. However, the pluralistic nature of attributions for MNS disorders was evident, with 45% of PHC workers also endorsing supernatural causes of MNS disorders (51.9% of diploma nurses).

The marked difference between degree and diploma level PHC workers with regard to knowledge about MNS disorders and psychotropic medications, and attitudes towards mental health care in PHC, has important implications for the scale-up of mental health care in Ethiopia. Diploma level nurses make up the majority of the PHC workforce (around three-quarters in this study) and are the cadre of health professional who are expected to deliver the mhGAP interventions in Ethiopia, including identification of MNS disorders and initiation of psychotropic medication where indicated. Brief, stand-alone training is unlikely to be sufficient to equip these health workers with sufficient clinical competence and confidence to deliver mental health care independently. As recommended by previous studies [35], ongoing training and supportive supervision by mental health professionals is likely to be essential. A previous study of the impact of brief mental health training of community health workers in India found that recognition of MNS disorders increased and faith in non-helpful pharmacological interventions (e.g. non-indicated vitamin injections or benzodiazepines) decreased, but that stigmatising beliefs were more resistant to intervention [36]. This further underscores the need for training courses to target known misconceptions and include personal testimonies with MNS disorders who have experienced mental health care [30].

Substantial logistical challenges to task sharing mental health care were evident from this study. If mental health care is going to be integrated successfully into PHC, structural problems such as lack of examination rooms, non-availability of medications for MNS disorders and the shortage of time for PHC workers to diagnosis and treat MNS disorders will need to be addressed. Provision of clinical guidelines and periodic supportive supervision by a mental health professional would be possible ways to support integration of mental health care into PHC, supported by evidence from previous studies [35]. However, in this study, the health centres were up to five hours travel from the Zonal town, where the psychiatric unit is located, limiting the feasibility of this approach without additional availability of specialist mental health workers [37]. Evaluation of an implemented model of task sharing mental health care in PHC in Ethiopia is underway and will yield important insights regarding acceptability and feasibility of this approach [38]. Other innovative approaches may also need to be considered in addition, or even as an alternative to, the task sharing approach with PHC workers. The biggest burden of mental health care in Ethiopia currently falls upon the family [11], with traditional and religious healers contributing to the care of people with severe mental illnesses such as psychosis. Capitalising on existing community expertise and interest in the problem of mental illness may be a more sustainable approach in the longer-term, for example, training up local mental health champions who would be linked to PHC and provide a focus for mental health care.

### Strengths and limitations

#### Limitations

Overall, this study indicates that there is support from frontline PHC workers for integrating mental health care into PHC as a way of facilitating early detection and intervention for mental health problems. However, the study did not include other relevant stakeholders within the health system, including people with mental health problems and their families, health facility managers, political leaders, high level officials and policy makers, or community-based health workers. Given that the PHC workers were aware that the study was being conducted by the Department of Psychiatry, Jimma University, and that the Federal Ministry of Health was advocating task sharing mental health in PHC, responses may have been subject to social desirability bias. As the PHC workers were not currently delivering mental health care, their responses were based on perceptions rather than actual experience of task sharing. The interpretation of PHC workers of some of the questions could have been different to that which was intended. To overcome this, future studies employing a more in-depth qualitative approach are warranted. The study was carried out in just one region of Ethiopia; however, the religious backgrounds of participating health care workers were representative of Ethiopia as a whole. Furthermore, we believe that the findings are likely to be generalisable to many low-income country settings which also contend with poor PHC infrastructure, low levels of mental health service coverage and limited availability of mental health specialists.

#### Strengths

The study was conducted across a predominantly rural area and included a representative sample of health staff and centres, ranging from relatively inaccessible facilities to those located in the Zonal town.

## Conclusions

The high level of acceptance of the need to decentralise mental health care to PHC by the health centre workers is a positive starting point, although the lower level of willingness to be actually involved in delivery of mental health care will be a challenge to implementation. The feasibility of integrating mental health into PHC in this sub-Saharan African setting is limited by important gaps in PHC worker knowledge and expectations regarding mental health care, coupled with health system constraints. In addition to clinically-based refresher mental health training, expansion of the specialist mental health workforce will be needed to support integration in practice.

## Abbreviations

HEW: Health extension worker; HIV: Human immunodeficiency virus; LMIC: Low- and middle-income country; mhGAP: Mental health gap action programme; MNS: Mental, neurological and substance use; PHC: Primary health care; WHO: World Health Organization.

## Competing interests

The authors declare that they have no competing interests.

## Authors’ contributions

MA, TB, MT and CH all contributed to the study design, MA conducted the study, supervised in the field by MT and TB. MA, MT and CH analysed the data. MA wrote the first draft of the manuscript. All authors contributed to interpretation of the study findings. All authors reviewed and approved the final manuscript prior to submission.

## Pre-publication history

The pre-publication history for this paper can be accessed here:

http://www.biomedcentral.com/1472-6963/14/113/prepub
